# Therapeutic Approach for Trigeminal Neuralgia: A Systematic Review

**DOI:** 10.3390/biomedicines11102606

**Published:** 2023-09-22

**Authors:** Muhammad Haseeb Rana, Abdul Ahad Ghaffar Khan, Imran Khalid, Muhammad Ishfaq, Mukhatar Ahmed Javali, Fawaz Abdul Hamid Baig, Mohammad Zahir Kota, Mohasin Abdul Khader, Mohammad Shahul Hameed, Sharaz Shaik, Gotam Das

**Affiliations:** 1 Department of Prosthodontics, College of Dentistry, King Khalid University, Abha 61421, Saudi Arabia; haseebrana80@gmail.com; 2 Department of Oral and Maxillofacial Surgery, College of Dentistry, King Khalid University, Abha 61421, Saudi Arabia; abahkhan@kku.edu.sa (A.A.G.K.); imajid@kku.edu.sa (I.K.); mishfaq@kku.edu.sa (M.I.); fbik@kku.edu.sa (F.A.H.B.); mqota@kku.edu.sa (M.Z.K.); 3 Department of Periodontics and Community Dental Sciences, College of Dentistry, King Khalid University, Abha 61421, Saudi Arabia; jmahmad@kku.edu.sa (M.A.J.); mabdulqader@kku.edu.sa (M.A.K.); 4 Department of Diagnostic Sciences and Oral Biology, College of Dentistry, King Khalid University, Abha 61421, Saudi Arabia; mohammad.shahul@gmail.com; 5 Department of Prosthetic Dentistry, Lenora Institute of Dental Sciences, Rajahmundry 533101, India; sharazshaik@gmail.com

**Keywords:** trigeminal neuralgia, pharmacological management, pain, neuralgia, drug therapy

## Abstract

This umbrella review aimed to determine the various drugs used to treat trigeminal neuralgia (TN) and to evaluate their efficacies as well as side effects by surveying previously published reviews. An online search was conducted using PubMed, CRD, EBSCO, Web of Science, Scopus, and the Cochrane Library with no limits on publication date or patients’ gender, age, and ethnicity. Reviews and meta-analyses of randomized controlled trials pertaining to drug therapy for TN, and other relevant review articles added from their reference lists, were evaluated. Rapid reviews, reviews published in languages other than English, and reviews of laboratory studies, case reports, and series were excluded. A total of 588 articles were initially collected; 127 full-text articles were evaluated after removing the duplicates and screening the titles and abstracts, and 11 articles were finally included in this study. Except for carbamazepine, most of the drugs had been inadequately studied. Carbamazepine and oxcarbazepine continue to be the first choice for medication for classical TN. Lamotrigine and baclofen can be regarded as second-line drugs to treat patients not responding to first-line medication or for patients having intolerable side effects from carbamazepine. Drug combinations using carbamazepine, baclofen, gabapentin, ropivacaine, tizanidine, and pimozide can yield satisfactory results and improve the tolerance to the treatment. Intravenous lidocaine can be used to treat acute exaggerations and botulinum toxin-A can be used in refractory cases. Proparacaine, dextromethorphan, and tocainide were reported to be inappropriate for treating TN. Anticonvulsants are successful in managing trigeminal neuralgia; nevertheless, there have been few studies with high levels of proof, making it challenging to compare or even combine their results in a statistically useful way. New research on other drugs, combination therapies, and newer formulations, such as vixotrigine, is awaited. There is conclusive evidence for the efficacy of pharmacological drugs in the treatment of TN.

## 1. Introduction

**Figure 1:**
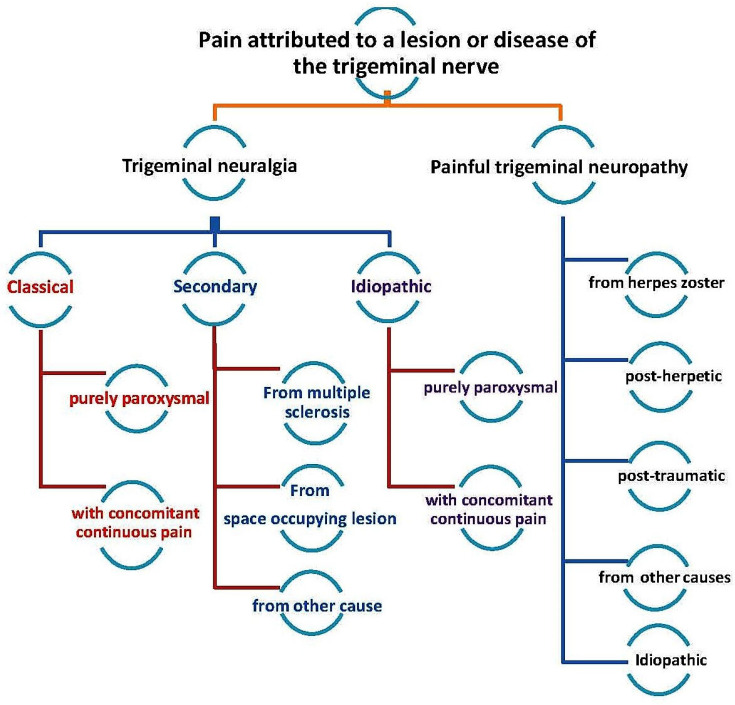
Classification of trigeminal nerve disorders, obtained from the study by Zhange et al., 2016 [3].

Trigeminal neuralgia (TN), also known as tic douloureux, is a disorder that affects the trigeminal nerve and is characterized by the presence of sudden, repetitive waves of pain that last from a few seconds to a few minutes [1]. The pain might be triggered by a sensory stimulus on the face, lips, or oral mucosa, or during certain functional movements of the face, and it is a debilitating chronic condition that occurs following injury or inflammation of the peripheral trigeminal nerve. As described in a recent review, peripheral and central nociceptive circuits are involved in neuropathic pain conditions that involve the trigeminal nerve (Bista and Imlach) [2]. TN is one of several disorders related to the trigeminal nerve [3] and is divided into three major types: classical, secondary, or idiopathic (Figure 1). The most common cause of TN, classical TN, is vascular compression of the trigeminal nerve at the root entry zone. Microvascular decompression (MVD) is the first-line treatment for classical TN. Secondary TN can be caused by tumors or vascular abnormalities in the posterior fossa, or it might be caused by multiple sclerosis. It is called idiopathic TN when no cause can be found [4,5]. The term paroxysm has been used to describe the sudden, unexpected, and short-lasting nature of the pain. However, some of TN patients experience concomitant continuous pain in the form of a dull ache in the same area as the paroxysmal pain [4]. Although the exact mechanism involved in the development of TN remains poorly understood, the potential mechanisms involve peripheral and central nociceptive circuit dysfunctions, which could lead to cross-excitation of an intact nerve by an injured nerve, neuro-glial interactions, alterations in the wirings within the CNS, loss of inhibitory control, disruptions in the functions of ion channels, and sensitization by inflammatory mediators (Bista and Imlach) [5].

TN can impact the psychological, emotional, and socioeconomic well-being of a patient. The treatment of this condition is often challenging, and the majority of the therapies focus on symptomatic relief to lessen the intensity and incidence of pain [4]. The therapeutic modalities are based on pharmacological, interventional, and alternative medicine. Pharmaceutical management is minimally invasive, and antiepileptics have mainly been used as first-line treatment [5]. Carbamazepine has been used to treat various types of neuropathic pain since the early 1960s; however, the side effects associated with this drug are a significant cause for concern [6]. Subsequently, several antiepileptic and other types of drugs have been used to treat neuropathic pain, including TN (Table 1).

Several studies on the efficacies of the various treatment modalities for TN have been published; additionally, systematic reviews of these interventional studies are available in the literature (Table 1). More than 30 pharmacological interventions have been used to treat TN. However, they have not been comprehensively reviewed in most of the systematic reviews, except for a few that included two or more drugs [5,9,56,57,58,59,60,61,62,63,64,65,66,67,68]. In 2019, the European Academy of Neurology published a survey of the developments in the diagnosis and treatment of TN and emphasized the necessity for further studies on the therapeutics for this condition [69]. Recent results indicate that TN behaves significantly differently than previously expected. The first reaction of patients treated with the various medication combinations was rather positive. Side effects were widespread with all the medicines, along with a reduced quality of life, which may lead to therapy discontinuation. However, drug resistance was uncommon, and fewer individuals required neurosurgery. This shows that the immediate response rate is higher than previously anticipated [44,59,66].

This is the first systematic review to methodically recognize, organize, classify, and summarize information about the efficacy of therapeutics for TN from several international review articles. This review aimed to identify and assess the available review articles, evaluate their quality, compile and compare their conclusions, and assess their merits for drawing the best evidence. The information from this review article can be used as an updated (2022-December) reference for health care providers and can contribute to evidence-based (empirical) decision making.

## 2. Methodology

This systematic review was performed and presented in accordance with the Preferred Reporting Items for Systematic Reviews and Meta-Analysis (PRISMA) 2009 statement. The systematic review protocol was registered in the Prospective Register of Systematic Reviews (PROSPERO), University of York, with the registration number CRD42023408948. The PICOS (participants, interventions, comparators, outcomes, and study design) method was used to determine the inclusion and exclusion criteria (Table 2).

Inclusion criteria: Reviews and meta-analysis of randomized controlled trials involving drug therapy for TN and other relevant reviews from articles included in their reference lists.

Exclusion criteria: Rapid reviews, reviews published in languages other than English, reviews of laboratory studies and uncontrolled trials, reviews of qualitative studies, and case reports/series.

Full-text screening: Full-text screening was conducted starting from the latest articles to the older ones. The drug(s) evaluated and the publication dates of the clinical trials included in each systematic review were noted. Reviews referring to the latest drug trials were included in our systematic review. Older articles were included only if they contained data that were not reported in the newer review articles. One or more systematic reviews were selected for each drug.

Search strategy and data collection process: Two independent reviewers conducted an online search, which was limited to reviews and meta-analyses published in the English language, regardless of the time of study and the gender, age, and ethnicity of the patients, using multiple databases (Table 3). The collection process ended on 1 December 2022.

**Figure 2:**
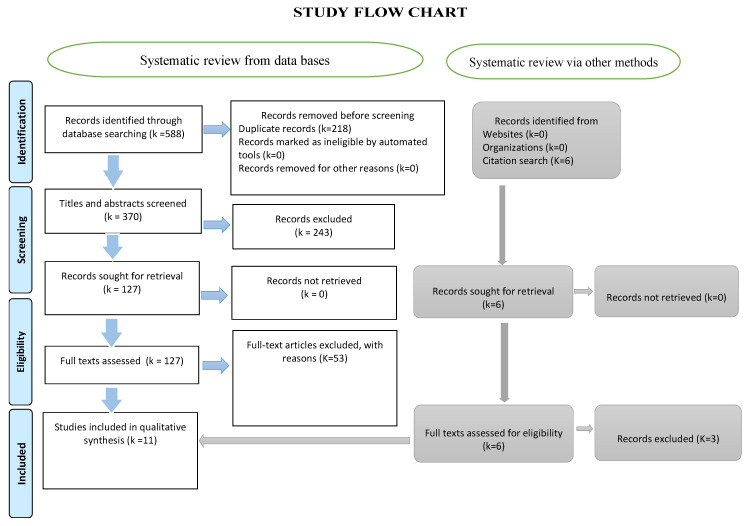
The PRISMA flow chart illustrating the various stages of the systematic review.

Data screening and retrieval: Two independent reviewers examined the segregated collection of titles and authors and excluded the duplicates. The titles and abstracts were evaluated and review articles were selected based on the aforementioned inclusion and exclusion criteria. The two independent selections of the two reviewers were matched, and discord was settled by a third reviewer. Only full-text articles were analyzed and the data were retrieved using a specially designed template (Figure 2). The elements included in the data retrieval form were as follows: intervention, control, authors, year of publication, reported level of evidence, the efficiency of intervention over control, adverse effects, study conclusions, and our comments.

### Data Synthesis

The full text of each article was reviewed and summaries were generated individually by two reviewers. The data were classified and the comments of each reviewer were added after verifying the reference cited in the included systematic review and the involved clinical trial. The synthesized data were discussed among all the reviewers and a consensus was reached.

## 3. Results

Out of the 588 titles initially selected, 370 were screened following the removal of duplicates. Subsequently, 127 full texts were thoroughly evaluated and 11 review articles were finally included in this study (Figure 2).

A total of 31 pharmacological interventions were evaluated. The articles were analyzed and the drugs were evaluated, and their efficacy, the level of evidence, the adverse effects, and the conclusions were summarized. Table 4 presents a compilation of the essential drugs used for the medical management of TN.

### 3.1. Additional Information about the Drugs Included in the Review Articles

#### 3.1.1. Carbamazepine and Oxcarbazepine

Carbamazepine and oxcarbazepine have been considered the first line of treatment for pain management [5,73]. The adverse effects of these two drugs are a cause for concern because they can affect the patient’s willingness to continue with the treatment. Oxcarbazepine has relatively fewer adverse effects than carbamazepine. Oxcarbazepine may be considered as the first-line medication for secondary TNs; however, patients with multiple sclerosis need to be monitored for the primary disease [5]. The significant side effects associated with carbamazepine necessitated a search for more efficacious and safe substitutes.

#### 3.1.2. Eslicarbazepine

Eslicarbazepine acetate is a third-generation antiepileptic drug that belongs to the dibenzazepine family, along with carbamazepine and oxcarbazepine. The most common side effects of this drug include dizziness, nausea, vomiting, and drowsiness. There is insufficient evidence about the benefits of this drug. A retrospective and open-label study reported the effectiveness of eslicarbazepine (dose, 200–1200 mg/day) for TN. However, it can be considered for the treatment of neuropathic pain, headaches, and cranial neuralgia in TN patients who do not respond to or tolerate the first-line treatments.

#### 3.1.3. Gabapentin

A meta-analysis of randomized controlled trials comprising a total of 1331 patients reported a comparable efficacy of gabapentin with carbamazepine, with considerably fewer side effects [8]. Gabapentin might be considered a second-line drug and used as refractory therapy for patients with TN. It presented with a superior life satisfaction index B over carbamazepine and was associated with significantly fewer adverse effects [8]. Nonetheless, the efficacy of gabapentinoids for the treatment of TN has not been sufficiently evaluated.

#### 3.1.4. Ropivacaine and Gabapentin

Ropivacaine is a long-acting amide local anesthetic. A single-blind, low-participant clinical trial evaluated the combined effect of ropivacaine (peripheral nerve block in the trigger zone) and gabapentin (oral administration) and reported that they induced analgesia and improved the quality of life of the patient [5,21,71]. Although ropivacaine alone demonstrated adequate analgesia, combination therapy with a low dose of gabapentin demonstrated superior overall results [71].

#### 3.1.5. Ropivacaine and Carbamazepine

Similarly, the combination of ropivacaine and carbamazepine was found to reduce the side effects and limitations of carbamazepine [4].

#### 3.1.6. Lamotrigine

Lamotrigine might be considered as a second-level drug for TN. The patient-reported adverse effects of lamotrigine (vertigo, nausea, visual impairment, and reduced neuromuscular control) are similar to those seen with carbamazepine and oxcarbazepine. Furthermore, it is associated with a high incidence of dermatological reactions, which can be reduced by regulating the dosage [5]. Lamotrigine can be used to treat secondary TN; however, it can aggravate the symptoms of multiple sclerosis, in some instances. Nonetheless, the levels of evidence with regard to this drug are generally low [5].

#### 3.1.7. Levetiracetam

Levetiracetam is an antiepileptic drug that is thought to act by targeting high-voltage, N-type calcium channels and the synaptic vesicle protein 2A, thereby hindering the conduction of impulses through the synapse. It has shown acceptable efficacy and tolerability in patients with TN [4]. Although reports suggest the efficacy of levetiracetam in TN, most of them were open-label, pilot, or short-term studies with inadequate numbers of participants and a dosage of about 3–4 g/day [4]. Hence, additional randomized and placebo-controlled trials are warranted to confirm its effects.

#### 3.1.8. Phenytoin

Phenytoin is a well-known antiepileptic drug used to manage seizures and reduce anxiety. It has been used to treat TN and other types of neuropathic pain and fibromyalgia in some instances. However, there is insufficient evidence to support its benefits in TN patients, and further qualitative randomized trials are required to confirm its effects.

#### 3.1.9. Pregabalin

Pregabalin is an analog of gamma-aminobutyric acid type B (GABA) and structurally similar to gabapentin. It has been used to treat neuropathic pain in some cases, but there is little evidence of its benefits in patients with TN. The side effects of this drug are less marked than those of other antiepileptic drugs. The combination of pregabalin and carbamazepine was found to be effective in the case of refractory trigeminal neuralgia [4].

#### 3.1.10. Topiramate

Topiramate is an antiepileptic used to treat seizures. A systematic review and meta-analysis [7] of all available randomized controlled trials indicated that the efficacy of topiramate was similar to that of carbamazepine, with acceptable patient tolerability. However, the studies included in the meta-analysis had low-grade evidence.

#### 3.1.11. Proparacaine

A review reported that ophthalmic anesthetic proparacaine drops (0.5%) did not significantly improve the symptoms of TN when compared to the placebo, thus indicating that it might not produce long-lasting effects [70]. Hence, based on current evidence, the use of this drug for TN can be ruled out.

#### 3.1.12. Dextromethorphan

The effect of the cough suppressant dextromethorphan was compared with that of lorazepam in one study [56]. However, it proved to be ineffective for the treatment of TN.

#### 3.1.13. Tizanidine

Low-grade evidence from a double-blinded cross-over study comprising 11 participants indicated adequate analgesia following the use of tizanidine when compared to that using a placebo [59]. However, an insufficient number of participants attained complete analgesia; moreover, the efficacy was lost after a few months. The patient-reported side effects, such as dizziness, drowsiness, and tiredness were not significant [59]. In another study, the side effects associated with tizanidine were less than those observed with carbamazepine; however, the clinical efficacy of this drug was also lower than that of carbamazepine. Moreover, the sample size was small, and the highest doses used in the study were 18 mg for tizanidine and 900 mg for carbamazepine [5]. The inefficiency of tizanidine in providing relief could be attributed to its low influence on the neural reaction of low-threshold mechanoreceptors, which possibly plays an essential role in the pathophysiology of TN [64].

#### 3.1.14. Pimozide

Pimozide is an antipsychotic drug and, in most studies, it appeared to be superior to carbamazepine in providing relief against noncompliant trigeminal neuralgia; however, the side effects, which included central nervous system disorders, hand shivers, and diminished cognition, were found to be significant [5]. Alternatively, Yang, F. et al. [60] reported that the effect of pimozide was inferior to the other seven drugs and placebo used for comparison in their study [60].

#### 3.1.15. Tocainide

The palliative outcome of the antiarrhythmic agent tocainide was comparable to that of carbamazepine [56]. However, the common side effects of this drug, which included nausea, paresthesia, and dermatological reactions, limited its use for the treatment of TN.

#### 3.1.16. Lidocaine

Lidocaine has been used in various forms: topical application (8%) in the oral mucosa; nasal spray; local infiltration in trigger zones; and intravenous infusion. The combination of lidocaine and ropivacaine, carbamazepine, or gabapentin has yielded better results than carbamazepine alone [60].

#### 3.1.17. Baclofen

Baclofen might be effective against TN secondary to multiple sclerosis; it relieves the spasticity observed in patients with multiple sclerosis. However, it can cause momentary drowsiness and muscular hypotonia. Furthermore, a sudden withdrawal of the drug could instigate convulsions and delirium. In one study, levorotatory baclofen was found to be exponentially effective and demonstrated reduced side effects when compared to racemic baclofen [59]; however, the study comprised a limited number of participants for short-term therapy.

Low-grade evidence from a study on baclofen and carbamazepine reported that baclofen alone was slightly superior to carbamazepine alone; the combination of both these drugs, however, was significantly superior [59]. Although the aforementioned study was double-blinded and randomized, the results can only be considered as low-grade evidence due to the limited sample size and a participant dropout rate of 30% [59].

#### 3.1.18. Sumatriptan

The subcutaneous injection of sumatriptan (3 mg) with oral administration (100 mg/day) yielded a significant improvement in analgesia in patients with TN and was sustained for a week after the termination of the treatment [70]. A random effects rankogram evaluation revealed that the effects of sumatriptan were superior to those of intranasal lidocaine, intravenous lidocaine, botulinum toxin, ophthalmic proparacaine, and a placebo [70].

#### 3.1.19. Botulinum Toxin A

Botulinum toxin type A is an exotoxin released by the Gram-positive bacterium *Clostridium botulinum*. It (intradermal or submucosal injection; dose range, 25–75U) was found to effectively produce analgesia in patients with TN [70]; however, the drug can affect the neuromuscular abilities of the involved muscles.

#### 3.1.20. Minocycline

Minocycline (MC) is a second-generation semi-synthetic tetracycline broad-spectrum antibiotic. It possesses a high lipophilicity, excellent tissue penetration, and a good anti-interference action. Nagpal Kalpana et al. investigated the central antinociceptive impact of nanoparticles loaded with minocycline hydrochloride and discovered that MC is uniquely central analgesic, suggesting that targeted nanoparticles could be employed to efficiently deliver the central antinociceptive action of TN. The findings imply that MC is an analgesic for TN, and the mechanism may be connected to a reduction in inflammatory factors [71].

#### 3.1.21. Novel Agents Used to Treat TN

Basimglurant is a strong, selective, and safe mGlu5-negative allosteric modulator with excellent oral bioavailability and a long half-life that supports once-daily treatment in humans. mGluR5 is essential for neurotransmitter release and neurotransmitter postsynaptic response in the central and peripheral neural systems. mGlu5 at synaptic and extrasynaptic sites can boost NMDA receptor activation by boosting NMDA currents. Inhibiting the downstream actions of the mGlu5 receptor may reduce NMDA function. Except in cases of prior application for serious depression, basimglurant may have favorable outcomes in the treatment of TN. In NCT05142228, the TN pain-related outcomes after the injection of aimovig (erenumab) under the skin are being evaluated. Aimovig is the first antibody therapy targeting the calcitonin gene-related peptide (CGRP) receptor, and it has FDA approval for migraine prevention, is well tolerated, and has a good safety profile. CGRP plays a pivotal role in the formation and maintenance of NP. This trial is an expanded application of aimovig (erenumab) to TN [72,73,74].

## 4. Discussion

Conservative pharmacological management is considered the first-line treatment for TN. Despite the considerable number of drugs used, the medical management of this condition remains challenging. The aim of this systematic review was to provide an overview of previously published systematic reviews on the pharmacological management of TN. Eleven articles encompassing 31 pharmacological interventions were evaluated (Table 4).

Carbamazepine and oxcarbazepine (daily recommended doses, 0.4–1.2 g and 0.9–1.8 g, respectively) have been considered the first line of treatment for pain management [5,75]. However, sleepiness, vertigo, nausea, double vision, and reduced neuromuscular control, as well as cognition issues, are the most frequent patient-reported side effects of these drugs, particularly in females [4]. Elevated levels of transaminases and hyponatremia are frequently observed in patients who receive higher doses of these medications. The presence of these side effects led to the search for other drugs that might prove beneficial for patients with TN.

One of the 11 articles included in the current systematic review indicated that there is a dearth of studies on the clinical efficacy of gabapentin as monotherapy for TN; only one study with high-grade evidence reported that ropivacaine (block) + gabapentin (oral administration) reduced pain and improved the quality of life of patients with TN [21,76].

Yang F. et al. recently published an NMA of eight different interventions for TN, but the primary treatment methods were not well defined [60]. They compared the response rates across studies, which might account for the discrepancy in findings with regard to pimozide, as indicated in Table 4. The comparison of response rates among studies might not yield clinically significant results because the treatment outcomes across these studies are not uniform. Another concern with regard to the study by Yang F. et al. was the advocacy of lidocaine as the first-line drug for TN. The studies included in the NMA had low-grade evidence in terms of the sample size and methodology used. Moreover, repeated applications due to the short duration of action of lidocaine could prove inconvenient for the patient. These findings were, subsequently, refuted by Steinberg in 2018 [9].

The review article by Alves et al. indicated that topiramate was not significantly different from the placebo in providing analgesia for patients with TN [56]; however, the finding was based on the results of one study only. Alternatively, the systematic review by Wang et al. in 2011 [6] reported that the efficacy of topiramate was initially similar to that of carbamazepine, but significantly improved after two months of therapy. However, the evidence levels of the studies included in their review were low. Therefore, further studies are needed to ascertain the clinical significance of the results. Nonetheless, we included the review by Alves et al. in the current study because it is the first and only systematic review to present the inefficacies of both dextromethorphan and proparacaine.

Gabapentin coupled with ropivacaine injections into trigger sites enhanced pain management and quality of life, while pregabalin was proven to be beneficial in TN patients after a year of follow-up [77,78,79]. Hu et al. recently conducted a systematic review of the therapeutic efficacy and safety of botulinum toxin type A (BTX-A) injections for TN and discovered a response in approximately 70–100% of patients, with mean pain intensity and frequency reduced by approximately 60–80% and no major adverse events reported [80].

Various combinations of carbamazepine, lamotrigine, gabapentin, and baclofen have been used for secondary TN [5]; however, most of them carry low-grade evidence. Concomitant continuous pain is the most difficult to treat, and although carbamazepine monotherapy might not prove useful, gabapentin and pregabalin might have beneficial effects in reducing this pain [5].

Sridharan and Sivaramakrishnan [70] published a meta-analysis consisting of botulinum toxin A-related studies with high-grade evidence in 2017. Subsequently, Rubis et al. [77] published a comprehensive review article in 2022 which covered most of the randomized control trials on botulinum toxin A. Nonetheless, further studies on the dosage, number and frequency of injections, and side effects are warranted.

Sumatriptan, intranasal lidocaine, and intravenous lidocaine were reported to provide adequate analgesia, but most of the studies included in the review articles presented low-grade evidence [70,76]. However, the treatment outcome measures and other reporting items were well defined.

The article by Di Stefano published in 2018 is the most comprehensive review published to date [6]. In addition to reviewing the published studies, the article provided information about the new interventions that were under trial and illustrated the side effects of the drugs more comprehensively than other articles.

## 5. Conclusions and Future Perspectives

Among the numerous interventions available for TN, pharmacological management is the most conservative approach and should be the preferred mode of treatment. Taken together, the findings of the 11 review articles included in this study indicate that carbamazepine and oxcarbazepine continue to be the first-line medication for classical TN; however, their side effects limit their use to some extent. Lamotrigine and baclofen can be regarded as second-line drugs in patients who do not respond to the first-line medications or present with intolerable side effects. Carbamazepine can be combined with newer drugs tested within the past few years, such as gabapentin, ropivacaine, tizanidine, or pimozide, to yield better or equivalent results and improve the tolerance to treatment. Intravenous lidocaine can be used to treat acute pain, whereas botulinum toxin A might be used as refractory therapy. Proparacaine, dextromethorphan, and tocainide were generally reported to be inappropriate for treating TN. Except for carbamazepine, most of the drugs reviewed in our article were inadequately studied. Thus, further studies are required to ascertain their clinical significance. Furthermore, new research on combination therapies and newer formulations, such as vixotrigine, is warranted.

## Figures and Tables

**Table 1:** The medications available for neuropathic pain.

Category	Drug	Category	Drug
Antiepileptic	Carbamazepine [6,7,8,9]	Anesthetics	Ketamine *, Methadone * [10]
	Clonazepam [11]		Lidocaine [12,13,14,15]
	Gabapentin [8,16,17,18,19,20,21]		
	Lacosamide [22]		Amitriptyline [23]
	Lamotrigine [17,24]	Antidepressants	
	Levetiracetam [25]		Duloxetine [14,26]
	Oxcarbazepine [27]		Milnacipran [28]
	Phenytoin [29]		Nortriptyline [30]
	Pregabalin [14,31,32,33]		Venlafaxine [34]
	Topiramate [7,17,35]		Desipramine [36]
	Valproic acid [37]	Antipsychotics [38]	
	Zonisamide [39]	Alpha-adrenoceptor stimulants	Clonidine [40]
		Opioids	Tramadol [41]
Herbal [42]			Buprenorphine [43,44]
NSAIDs [45]	Paracetamol [46]		Fentanyl [47]
Miscellaneous	Cannabis [48]		Hydromorphone [33,49]
	Capsaicin [50,51]		Methadone [52]
			Morphine [53]
			Oxycodone [54,55]

* NMDA antagonists.

**Table 2:** PICOS statement for the study.

Participants	Intervention	Control	Primary Outcomes	Secondary Outcomes	Study Design
Patients who are undergoing/underwent treatment for TN, irrespective of age, gender, and ethnicity.	All drugs used for the treatment of TN, irrespective of the route of administration (Table 1).	Pain relief with orthodox analgesics/opioids/placebo/another active drug.	1. Intensity of pain quantitatively assessed using the visual analog scale, numerical rating scale. 2. Incidence of painful episodes.	1. Therapeutic success based on the subjects’ reporting. 2. Quality of life measure. 3. Adverse effects.	Reviews and meta-analysis.

**Table 3:** The search strategy.

Interface	Database	Search Query	Article Output
National Library of Medicine	PubMed	Search: (trigeminal neuralgia) AND (pharmacological) AND (meta-analysis[Filter) OR review (Filter) OR (trigeminal neuralgia) AND (drug) AND (meta-analysis) Filter OR review (Filter) OR (trigeminal neuralgia) AND (medical) AND meta-analysis (Filter) OR review (Filter) OR (tic douloureux) AND (pharmacological) AND meta-analysis (Filter) OR review (Filter) OR (tic douloureux) AND (drug) AND meta-analysis (Filter) OR review (Filter) OR (tic douloureux) AND (medical) AND meta-analysis (Filter) OR review (Filter) Filters: Meta-Analysis, Review	85
CRD database center for reviews and dissemination (available till 2015)	DARE (Database of Abstracts of Reviews of Effects), NHS EED (National Health Services Economic Evaluation Database), and HTA (Health Technology Assessment)	Trigeminal neuralgia (or) tic douloureux AND pharmacological OR Trigeminal neuralgia (or) tic douloureux AND drug OR Trigeminal neuralgia (or) tic douloureux AND medical	25
EBSCO	Academic Search Ultimate, CINAH, MEDLINE, and Dentistry & Oral Sciences Source	“Trigeminal neuralgia OR tic douloureux AND systematic review AND pharmacological Scholarly (Peer Reviewed0 Journals AND Apply equivalent subjects Limiters– Scholarly [Peer Reviewed] Journals Expanders– Apply equivalent subjects Narrow by Subject Thesaurus: pregabalin, placebos, phenytoin, nonopioid analgesics nerve block, medical marijuana, local anesthetics, duloxetine, clonidine, baclofen, anticonvulsants, amitriptyline, indomethacin, hyponatremia, gabapentin, drug therapy, combination drug therapy, antidepressants, sodium channel blockers, pain management, lidocaine, lamotrigine, carbamazepine, therapeutics, trigeminal neuralgia. Narrow by Language: English Search modes– Find all my search terms	83
Web of Science	SCI-Expanded, SSCI, A&HCI, CPCI-S, CPCI-SSH, ESCI, and CCR-Expanded	TS = Trigeminal neuralgia OR tic douloureux AND AB = pharmacological TS = [Trigeminal neuralgia * OR tic douloureux AND AB = drug TS = Trigeminal neuralgia * OR tic douloureux AND AB = medical LANGUAGE: [English]; DOCUMENT TYPES: [Review] IC Timespan = All years	235
Scopus	Scopus	ALL “Trigeminal neuralgia” and TITLE-ABS “pharmacological” and d TITLE-ABS [metanalysis] OR [ALL [“Trigeminal neuralgia”] AND TITLE-ABS [“drug”] AND d TITLE-ABS [metanalysis] OR [ALL [“Trigeminal neuralgia”] AND TITLE-ABS [“medical”] AND d TITLE-ABS [metanalysis] OR ALL [“Trigeminal neuralgia”] and TITLE-ABS [“pharmacological”] AND d TITLE-ABS [systematic AND review] OR ALL [“Trigeminal neuralgia”] AND TITLE-ABS [“drug”] and d TITLE-ABS [systematic AND review] OR ALL [“Trigeminal neuralgia”] and TITLE-ABS [“medical”] AND d TITLE-ABS [systematic AND review]]]	103
Cochrane Library	Cochrane Database of Systematic Reviews	Trigeminal neuralgia [or] tic douloureux AND pharmacological OR Trigeminal neuralgia [or] tic douloureux AND drug OR Trigeminal neuralgia [or] tic douloureux AND medical	57
TOTAL			588

**Table 4:** Data collected from the 11 articles included in this review.

Drug	Control	Authors—Year—Ref	Reported Level of Evidence	Efficacy of the Drug over Control	Adverse Effects	Study Conclusions	Our Comments
Carbamazepine	Placebo	Yang, F. et al., 2018 [60]; Di Stefano, G. et al., 2018 [5]	High	Yes	Headache, reduced neuromuscular coordination, vertigo, sleepiness, nausea and vomiting, nephrosis, arrhythmias, constipation, dermatological reactions [68]	While carbamazepine and oxcarbazepine are successful in most patients, the side effects may discourage the patients from continuing the treatment, especially older adults.	Carbamazepine and oxcarbazepine continue to be the first drug of choice for trigeminal neuralgia. However, the incidence of side effects in the patients needs to be monitored, particularly in elderly patients.
	2. Lamotrigine	Yang, F. et al., 2018 [60]	Based on network meta-analysis (NMA)	Yes			
Oxcarbazepine	Placebo	Yang, F. et al., 2018 [60]	Based on NMA	Yes	Similar to but milder than that of oxcarbazepine [5]	Oxcarbazepine has lesser drug interactions than carbamazepine.	Carbamazepine and oxcarbazepine continue to be the first drug of choice for trigeminal neuralgia. However, the incidence of side effects in the patients needs to be monitored, particularly in elderly patients.
	2. Carbamazepine	Di Stefano, G. et al., 2018 [5]	Low	Similar	Safer than carbamazepine	Effective in classical TN. However, the efficacy is diminished in continuous concomitant pain.	The dissimilarity in the conclusions is due to differences in the source of the data. The Di Stefano conclusion was based on one randomized controlled trial, whereas the review by Yang F. et al. included two studies, one double-blind RCT and one retrospective study.
		Yang, F. et al., 2018 [60]	Based on NMA	No	Safer than carbamazepine	Oxcarbazepine can be chosen for fewer drug interactions and better tolerability.	
	3. Tizanidine	Yang, F. et al., 2018 [60]	Based on NMA	Yes	Not mentioned	Not mentioned.	
Eslicarbazepine	None	Di Stefano, G. et al., 2018 [5]	Low: retrospective, open-label, low participant count	Yes	Hyponatremia	Both the American Academy of Neurology Society and the European Federation of Neurological Society (AAN-EFNS) neither endorse nor refute the use of this drug in secondary TN.	It may be helpful in secondary TN and refractory TN. Needs further studies.
Gabapentin	Carbamazepine	Di Stefano, G. et al., 2018; Yaun et al., 2016 [5,8]	Low: methodological deficiencies	Similar	Sleepiness, vertigo, diminished neuromuscular control, fatigue diplopia, hand tremor; the side effects are lower than those observed with carbamazepine; negligible drug interactions [21]	The superiority of gabapentin over carbamazepine could not be determined. One of the second-line drugs for secondary TN [5].	The data were deficient in determining the efficacy. Thus, additional studies are necessary.
	2. Oxcarbazepine	Ta, P.C.P. et al., 2019 [21]	Low: methodological deficiencies and small participant size, short duration	No	A comparison of side effects with oxcarbazepine is not included in the study	Though gabapentin was effective, it was not superior to oxcarbazepine [21]. Gabapentin could be useful in relieving concomitant continuous pain. Can be used in combination with carbamazepine or oxcarbazepine.	Additional methodologically sound randomized controlled trials are needed.
Ropivacaine + gabapentin	Gabapentin alone	Ta, P.C.P. et al., 2019 [21]; Di Stefano, G. et al., 2018 [5]	Methodologically sound, but low participant count; to date, only one study is available	Yes	Not specified but the side effects were less than gabapentin alone	Effective even after 11 months in many cases. Analgesia, quality of life scores, and oral functions were enhanced.	Though the study was well designed, further studies are needed on a sizable population.
Ropivacaine (nerve block) + carbamazepine (oral)	Carbamazepine	Di Stefano, G. et al., 2018 [5]	Methodologically sound but low participant count; to date, only one study is available	Yes	Less than carbamazepine	Substantial reduction in the pain intensity and daily incidences despite reducing the daily dose of carbamazepine.	The combination therapy can reduce the side effects and limitations of carbamazepine.
Lamotrigine	Placebo	Yang, F. et al., 2018 [60]	NMA	Yes	Dermatological reactions, vertigo, headache, constipation, nausea, dysgeusia, mental distress [68]	One of the second-line drugs for secondary TN [5]. The EFNS 2010 advocated the use of lamotrigine for intractable trigeminal neuralgia [70].	The EFNS advocates lamotrigine for intractable cases.
	2. Carbamazepine	Yang, F. et al., 2018 [60]; Di Stefano et al., 2017 [5]	NMA	No	Less than carbamazepine [68]	Not discussed.	
	3. Tizanidine	Yang, F. et al., 2018 [60]	NMA	No	Not mentioned	Not discussed.	
Lamotrigine + carbamazepine or phenytoin	Placebo	Di Stefano, G. et al., 2018 [5]	Low	Yes, slightly more effective	Not mentioned in the study	Not discussed.	
Levetiracetam	Low: open-label, unspecified control/observational trial.	Di Stefano, G. et al., 2018 [5]	Low	Yes	Not mentioned in the study	Not discussed.	It can be used in classical TN and refractory TN
Phenytoin	Low: there were no methodologically sound studies to date.	Di Stefano, G. et al., 2018 [5]	None	Yes?	Not mentioned in the study	Not discussed.	Phenytoin was the foremost drug used for TN, yielding encouraging results. However, additional methodologically sound, randomized controlled trials are needed.
Pregabalin	Not mentioned in the study	Di Stefano, G. et al., 2018 [5]	Low	Yes	Not mentioned in the study	Pregabalin may be effective in relieving concomitant continuous pain. Combination with carbamazepine or oxcarbazepine is recommended.	Additional methodologically sound, randomized controlled trials are needed.
	2. Lamotrigine	Sridharan and Sivaramakrishnan 2016 [70]	Low	Yes; according to the RCT	Side effects of pregabalin are less than lamotrigine	The reviewers could not perform a meta-analysis on this comparison.	Further studies on larger sample sizes are needed.
Pregabalin + carbamazepine	Lamotrigine + carbamazepine	Di Stefano, G. et al., 2018 [5]	Low: open-label, low participant count	Similar	Pregabalin produced fewer adverse effects than lamotrigine	The combination can be used in refractory TN.	Effective in the case of refractory trigeminal neuralgia.
Topiramate	Placebo	Alves TCA 2004 [56]		No	Nausea, diarrhea, tiredness, drowsiness, and diminished cognition; however, no patient dropped out [56]	One of the second-line drugs for secondary TN [5].	The findings in the review by Alves et al. are from one study only. The later studies reported positive results.
	2. Carbamazepine	Wang et al., 2011 [7]	From NMA. Low: quality deficient in methods and topography of the study	Yes [after 2 months]	Similar	The initial efficacy and sides effects were similar.	Further studies are needed.
Proparacaine	Placebo	Yang, F. et al., 2018 [60]; Zhang 2013 [59]; Alves TCA 2004 [56]	High (low risk of bias)	No	Negligible [68]	Proparacaine might not be producing long-lasting actions. A combination with lidocaine could be investigated [56].	Based on the evidence, proparacaine could be ruled out for use in TN.
	2. Carbamazepine	Yang, F. et al., 2018 [60]	NMA	No	Not mentioned	Proparacaine produced inferior results to placebo.	
	3. Lidocaine	Yang, F. et al., 2018 [60]	NMA	No			
Dextromethorphan	Lorazepam	Alves TCA 2004 [56]		No	Diminished cognition, dizziness, and ataxia; no patient dropouts [56]	Many patients under dextromethorphan reported a rise in pain.	The drug was proven to be inefficient.
Tizanidine	Placebo	Yang, F. et al., 2018 [60]	NMA	Yes	Vertigo and tiredness [68]	Not discussed.	Extensive sample-sized studies are required.
	2. Carbamazepine	Yang, F. et al., 2018 [60]; Di Stefano, G. et al., 2018 [5]; Zhang et al. 2013 [59]	NMA (Yang et al.); small sample size	No/No significant difference	Side effects of tizanidine are fewer than those of carbamazepine [59]	Extensive sample-sized studies are required.	
Pimozide	Placebo	Yang, F. et al., 2018 [60]	NMA	No	Physical and psychological impedance, hand shivers, diminishing cognition [68]	Not discussed.	The finding was based on NMA. The authors, however, did not discuss the disagreement with other studies.
	2. Carbamazepine	Yang, F. et al., 2018 [60]	NMA	No	According to this NMA, the effect of pimozide was inferior to those of the other drugs compared, including the placebo	Not discussed.	Based on NMA, the findings contradict those of Di Stefano, G. et al., 2018 and Zhang et al., 2013. However, the review did not discuss the conflict.
		Alves TCA 2004 [56]; Di Stefano, G. et al., 2018 [5]; Zhang et al., 2013 [59]	Low	Yes	The side effects of pimozide were worse than carbamazepine [68]	One review advocates lamotrigine and pimozide as second-line drugs for refractory cases [56].	The findings are contradictory to those of Yang F 2018. Lamotrigine and pimozide can be considered as one of the second-line drugs for intractable cases.
Tocainide	Carbamazepine	Alves TCA 2004 [56]; Di Stefano, G. et al., 2018 [5]; Zhang et al., 2013 [59]	Low	No/Inconclusive	Nausea, paresthesia, and dermatological reactions [68]	Significant side effects limited its use [5,59].	Based on the evidence, the drug is not beneficial for the treatment of TN.
Lidocaine 8% topical	Placebo	Yang, F. et al., 2018 [60]; Di Stefano, G. et al., 2018 [5]; Sridharan et al., 2016 [70]	NMA, low participant count	Yes	Transient local irritation [68]	The analgesia lasted for 3–4 h after application.	The studies have several shortcomings; the results need to be verified with further studies.
	2. Carbamazepine	Yang, F. et al., 2018 [60]	NMA	Yes		Not discussed.	
	3. Oxcarbazepine	Yang, F. et al., 2018 [60]	NMA	Yes			
	4. Tizanidine	Yang, F. et al., 2018 [60]	NMA	Yes			
Lidocaine (i.v. 5 mg/kg over 60 min)	Placebo	Di Stefano, G. et al., 2018 [5]; Sridharan and Sivaramakrishnan 2016 [70]	Low participant count	Yes	The side effects were mild and included sleepiness, xerostomia, vertigo, and headache	The analgesia lasted for 24 h.	This technique can be used in acute exacerbations and refractory cases.
Baclofen	Placebo	Di Stefano, G. et al., 2018 [5]	Low: low participant count and treatment duration	Yes	Diminished neuromuscular control, tiredness, and nausea [68]	The studies have several shortcomings; hence, the findings should be carefully inferred.	It may be beneficial in refractory TN. Further studies are needed to ascertain the findings.
Baclofen + carbamazepine or phenytoin	Placebo	Di Stefano, G. et al., 2017 [71]	Low	Yes	Remissions may occur		It may be beneficial in refractory TN.
Sumatriptan (subcutaneous inj. 3 mg + oral 50 mg) twice daily	Placebo	Di Stefano, G. et al., 2018 [5]; Sridharan and Sivaramakrishnan 2016 [70]	Low [70]	Yes	Tiredness and nausea [68] It is not suggested for long-standing prescription due to triptan-related side effects [5].	Sridharan and Sivaramakrishnan advocated this as the best drug for intractable TN [70].	Further studies are needed to ascertain the benefits and possibility of lowering the dose.
	2. Botulinum toxin A	Sridharan and Sivaramakrishnan 2016 [70]	Low	Yes			
Botulinum toxin A Subcutaneous/ Dose: 10 IU to 75 IU	Placebo	Rubies et al., 2020 [72]; Sridharan and Sivaramakrishnan 2016 [70]	Unclear risk of bias	Yes	Induced facial asymmetry, headaches, hematoma, local irritation, and pain [68]	It may be given intramuscularly for the mandibular nerve. The side effects usually disappear within a week.	It is a favorable option in patients with intractable TN.

## Data Availability

The data presented in this study are available on request from the corresponding author.

## References

[1] Adams R.D. (1997). Principles of Neurology.

[2] Bista P., Imlach W.L. (2019). Pathological Mechanisms and Therapeutic Targets for Trigeminal Neuropathic Pain. Medicines.

[3] Zhang Y., Kong Q., Chen J., Li L., Wang D., Zhou J. (2016). International Classification of Headache Disorders 3rd edition beta-based field testing of vestibular migraine in China: Demographic, clinical characteristics, audiometric findings and diagnosis statues. Cephalalgia.

[4] Eide P.K. (2022). Familial occurrence of classical and idiopathic trigeminal neuralgia. J. Neurol. Sci..

[5] Moore D., Chong M.S., Shetty A., Zakrzewska J.M. (2019). A systematic review of rescue analgesic strategies in acute exacerbations of primary trigeminal neuralgia. Br. J. Anaesth..

[6] Di Stefano G., Truini A., Cruccu G. (2018). Current and Innovative Pharmacological Options to Treat Typical and Atypical Trigeminal Neuralgia. Drugs.

[7] Wiffen Philip J., Derry S., Moore R.A., KalsoEija A. (2014). Carbamazepine for chronic neuropathic pain and fibromyalgia in adults. Cochrane Database Syst. Rev..

[8] Wang Q.P., Bai M. (2011). Topiramate versus carbamazepine for the treatment of classical trigeminal neuralgia: A meta-analysis. CNS Drugs.

[9] Yuan M., Zhou H.Y., Xiao Z.L., Wang W., Li X.L., Chen S.J., Yin X.-P., Xu L.-J. (2016). Efficacy and Safety of Gabapentin vs. Carbamazepine in the Treatment of Trigeminal Neuralgia: A Meta-Analysis. Pain Pract..

[10] Steinberg D.I. (2018). Review: In trigeminal neuralgia, carbamazepine, botulinum toxin type A, or lidocaine improve response rate vs placebo. ACP J. Club..

[11] Aiyer R., Mehta N., Gungor S., Gulati A. (2018). A Systematic Review of NMDA Receptor Antagonists for Treatment of Neuropathic Pain in Clinical Practice. Clin. J. Pain..

[12] Corrigan R., Derry S., Wiffen P.J., Moore R.A. (2012). Clonazepam for neuropathic pain and fibromyalgia in adults. Cochrane Database Syst. Rev..

[13] de León-Casasola O.A., Mayoral V. (2016). The topical 5% lidocaine medicated plaster in localized neuropathic pain: A reappraisal of the clinical evidence. J. Pain. Res..

[14] Derry S., Wiffen P.J., Moore R.A., Quinlan J. (2014). Topical lidocaine for neuropathic pain in adults. Cochrane Database Syst. Rev..

[15] Plested M., Budhia S., Gabriel Z. (2010). Pregabalin, the lidocaine plaster and duloxetine in patients with refractory neuropathic pain: A systematic review. BMC Neurol..

[16] Masic D., Liang E., Long C., Sterk E.J., Barbas B., Rech M.A. (2018). Intravenous Lidocaine for Acute Pain: A Systematic Review. Pharmacotherapy.

[17] Shaheen A., Alam S.M., Ahmad A., Khan M. (2019). Clinical efficacy and tolerability of Gabapentinoids with current prescription patterns in patients with Neuropathic pain. Pak. J. Med. Sci..

[18] Raj Paudel K., Bhattacharya S.K., Rauniar G.P., Das B.P. (2011). Comparison of antinociceptive effect of the antiepileptic drug gabapentin to that of various dosage combinations of gabapentin with lamotrigine and topiramate in mice and rats. J. Neuro. Rural. Pract..

[19] Banday M., Syed Sameer A., Farhat S., Aziz R. (2013). Gabapentin: A pharmacotherapeutic panacea. Int. J. Pharm. Pharm. Sci..

[20] Wiffen P.J., Derry S., Bell R.F., Rice A.S.C., Tölle T.R., Phillips T., Moore R.A. (2017). Gabapentin for chronic neuropathic pain in adults. Cochrane Database Syst. Rev..

[21] Moore R.A., Wiffen P.J., Derry S., Rice A.S.C. (2014). Gabapentin for chronic neuropathic pain and fibromyalgia in adults. Cochrane Database Syst. Rev..

[22] Ta P.C.P., Dinh H.Q., Nguyen K., Lin S., Ong Y.L., Ariyawardana A. (2019). Efficacy of gabapentin in the treatment of trigeminal neuralgia: A systematic review of randomized controlled trials. J. Investig. Clin. Dent..

[23] Hearn L., Derry S., Moore R.A. (2012). Lacosamide for neuropathic pain and fibromyalgia in adults. Cochrane Database Syst. Rev..

[24] Moore R.A., Derry S., Aldington D., Cole P., Wiffen P.J. (2015). Amitriptyline for neuropathic pain in adults. Cochrane Database Syst. Rev..

[25] Wiffen P.J., Derry S., Moore R.A. (2013). Lamotrigine for chronic neuropathic pain and fibromyalgia in adults. Cochrane Database Syst. Rev..

[26] Wiffen P.J., Derry S., Moore R.A., Lunn M.P.T. (2014). Levetiracetam for neuropathic pain in adults. Cochrane Database Syst. Rev..

[27] Lunn M.P.T., Hughes R.A.C., Wiffen P.J. (2014). Duloxetine for treating painful neuropathy, chronic pain or fibromyalgia. Cochrane Database Syst. Rev..

[28] Zhou M., Chen N., He L., Yang M., Zhu C., Wu F. (2017). Oxcarbazepine for neuropathic pain. Cochrane Database Syst. Rev..

[29] Derry S., Phillips T., Moore R.A., Wiffen P.J. (2015). Milnacipran for neuropathic pain in adults. Cochrane Database Syst. Rev..

[30] Birse F., Derry S., Moore R.A. (2012). Phenytoin for neuropathic pain and fibromyalgia in adults. Cochrane Database Syst. Rev..

[31] Derry S., Wiffen P.J., Aldington D., Moore R.A. (2015). Nortriptyline for neuropathic pain in adults. Cochrane Database Syst. Rev..

[32] Blanco Tarrio E., GálvezMateos R., Zamorano Bayarri E., López Gómez V., Pérez Páramo M. (2013). Effectiveness of Pregabalin as Monotherapy or Combination Therapy for Neuropathic Pain in Patients Unresponsive to Previous Treatments in a Spanish Primary Care Setting. Clin. Drug Investig..

[33] Derry S., Bell R.F., Straube S., Wiffen P.J., Aldington D., Moore R.A. (2019). Pregabalin for neuropathic pain in adults. Cochrane Database Syst. Rev..

[34] Dauri M., Lazzari M., Casali M., Tufaro G., Sabato E., Sabato A. (2014). Long-Term Efficacy of OROS Hydromorphone Combined with Pregabalin for Chronic Non-Cancer Neuropathic Pain. Clin. Drug Investig..

[35] Gallagher H.C., Gallagher R.M., Butler M., Buggy D.J., Henman M.C. (2015). Venlafaxine for neuropathic pain in adults. Cochrane Database Syst. Rev..

[36] Wiffen P.J., Derry S., Lunn M.P.T., Moore R.A. (2013). Topiramate for neuropathic pain and fibromyalgia in adults. Cochrane Database Syst. Rev..

[37] Hearn L., Moore R.A., Derry S., Wiffen P.J., Phillips T. (2014). Desipramine for neuropathic pain in adults. Cochrane Database Syst. Rev..

[38] Gill D., Derry S., Wiffen P.J., Moore R.A. (2011). Valproic acid and sodium valproate for neuropathic pain and fibromyalgia in adults. Cochrane Database Syst. Rev..

[39] Seidel S., Aigner M., Ossege M., Pernicka E., Wildner B., Sycha T. (2013). Antipsychotics for acute and chronic pain in adults. Cochrane Database Syst. Rev..

[40] Moore R.A., Wiffen P.J., Derry S., Lunn M.P. (2015). Zonisamide for neuropathic pain in adults. Cochrane Database Syst. Rev..

[41] Wrzosek A., Woron J., Dobrogowski J., Jakowicka-Wordliczek J., Wordliczek J. (2015). Topical clonidine for neuropathic pain. Cochrane Database Syst. Rev..

[42] Duehmke R.M., Derry S., Wiffen P.J., Bell R.F., Aldington D., Moore R.A. (2017). Tramadol for neuropathic pain in adults. Cochrane Database Syst. Rev..

[43] Boyd A., Bleakley C., Hurley D.A., Gill C., Hannon-Fletcher M., Bell P., McDonough S. (2019). Herbal medicinal products or preparations for neuropathic pain. Cochrane Database Syst. Rev..

[44] Aiyer R., Gulati A., Gungor S., Bhatia A., Mehta N. (2018). Treatment of chronic pain with various buprenorphine formulations: A systematic review of clinical studies. Anesth. Analg..

[45] Wiffen P.J., Derry S., Moore R.A., Stannard C., Aldington D., Cole P., Knaggs R. (2015). Buprenorphine for neuropathic pain in adults. Cochrane Database Syst. Rev..

[46] Moore R.A., Chi C.C., Wiffen P.J., Derry S., Rice A.S. (2015). Oral nonsteroidal anti-inflammatory drugs for neuropathic pain. Cochrane Database Syst. Rev..

[47] Wiffen P.J., Knaggs R., Derry S., Cole P., Phillips T., Moore R.A. (2016). Paracetamol [acetaminophen] with or without codeine or dihydrocodeine for neuropathic pain in adults. Cochrane Database Syst. Rev..

[48] Derry S., Stannard C., Cole P., Wiffen P.J., Knaggs R., Aldington D., Moore R.A. (2016). Fentanyl for neuropathic pain in adults. Cochrane Database Syst. Rev..

[49] Mücke M., Phillips T., Radbruch L., Petzke F., Häuser W. (2018). Cannabis-based medicines for chronic neuropathic pain in adults. Cochrane Database Syst. Rev..

[50] Stannard C., Gaskell H., Derry S., Aldington D., Cole P., Cooper T.E., Knaggs R., Wiffen P.J., Moore R.A. (2016). Hydromorphone for neuropathic pain in adults. Cochrane Database Syst. Rev..

[51] Derry S., Moore R.A. (2012). Topical capsaicin [low concentration] for chronic neuropathic pain in adults. Cochrane Database Syst. Rev..

[52] Derry S., Rice A.S.C., Cole P., Tan T., Moore R.A. (2017). Topical capsaicin [high concentration] for chronic neuropathic pain in adults. Cochrane Database Syst. Rev..

[53] McNicol E.D., Ferguson M.C., Schumann R. (2017). Methadone for neuropathic pain in adults. Cochrane Database Syst. Rev..

[54] Cooper T.E., Chen J., Wiffen P.J., Derry S., Carr D.B., Aldington D., Cole P., Moore R.A. (2017). Morphine for chronic neuropathic pain in adults. Cochrane Database Syst. Rev..

[55] Gaskell H., Derry S., Stannard C., Moore R.A. (2016). Oxycodone for neuropathic pain in adults. Cochrane Database Syst. Rev..

[56] Gaskell H., Moore R.A., Derry S., Stannard C. (2014). Oxycodone for neuropathic pain and fibromyalgia in adults. Cochrane Database Syst. Rev..

[57] Azevedo Alves T.C., Santos Azevedo G., Santiago de Carvalho E. (2004). Pharmacological treatment of trigeminal neuralgia: Systematic review and metanalysis. Revista Anest..

[58] Finnerup N.B., Sindrup S.H., Jensen T.S. (2010). The evidence for pharmacological treatment of neuropathic pain. Pain.

[59] Jawahar R., Oh U., Yang S., Lapane K.L. (2013). A systematic review of pharmacological pain management in multiple sclerosis. Drugs.

[60] Zhang J., Yang M., Zhou M., He L., Chen N., Zakrzewska J.M. (2013). Non-antiepileptic drugs for trigeminal neuralgia. Cochrane Database Syst. Rev..

[61] Yang F., Lin Q., Dong L., Gao X., Zhang S. (2018). Efficacy of 8 different drug treatments for patients with trigeminal neuralgia: A network meta-analysis. Clin. J. Pain..

[62] Vadalouca A., Siafaka I., Argyra E., Vrachnou E.V.I., Moka E. (2006). Therapeutic Management of Chronic Neuropathic Pain. Ann. N. Y. Acad. Sci..

[63] Murnion B.P. (2018). Neuropathic pain: Current definition and review of drug treatment. Aust. Prescr..

[64] Henze T., Rieckmann P., Toyka K.V. (2006). Symptomatic treatment of multiple sclerosis: Multiple Sclerosis Therapy Consensus Group [MSTCG] of the German Multiple Sclerosis Society. Euro Neuro.

[65] Chole R., Patil R., Degwekar S.S., Bhowate R.R. (2007). Drug treatment of trigeminal neuralgia: A systematic review of the literature. J. Oral Maxillofac. Surg..

[66] Chaparro L.E., Wiffen P.J., Moore R.A., Gilron I. (2012). Combination pharmacotherapy for the treatment of neuropathic pain in adults. Cochrane Database Syst. Rev..

[67] Benoliel R., Zini A., Khan J., Almoznino G., Sharav Y., Haviv Y. (2016). Trigeminal neuralgia [part II]: Factors affecting early pharmacotherapeutic outcome. Cephalalgia.

[68] Backonja M.-M., Serra J. (2004). REVIEW PAPERS Pharmacologic Management Part 1: Better-Studied Neuropathic Pain Diseases. Pain Med..

[69] Oomens M., Forouzanfar T. (2015). Pharmaceutical Management of Trigeminal Neuralgia in the Elderly. Drugs Aging.

[70] Bendtsen L., Zakrzewska J.M., Abbott J., Braschinsky M., Di Stefano G., Donnet A., Eide P.K., Leal P.R.L., Maarbjerg S., May A. (2019). European Academy of Neurology guideline on trigeminal neuralgia. Eur. J. Neurol..

[71] Gagpal K., Singh S.K., Mishra D.N. (2015). Minocycline encapsulated chitosan nanoparticles for central antinociceptive activity. Int. J. Biol. Macromol..

[72] Papadakis M.A., McPhee S.J. (2023). Trigeminal Neuralgia.

[73] Du Z., Zhang J., Han X., Yu W., Gu X. (2023). Potential novel therapeutic strategies for neuropathic pain. Front. Mol. Neurosci..

[74] Katsuki M., Kawamura S., Kashiwagi K., Tachikawa S., Koh A. (2023). Erenumab in the treatment of comorbid trigeminal neuralgia in patients with migraine. Cureus.

[75] Sridharan K., Sivaramakrishnan G. (2017). Interventions for Refractory Trigeminal Neuralgia: A Bayesian Mixed Treatment Comparison Network Meta-Analysis of Randomized Controlled Clinical Trials. Clin. Drug Investig..

[76] Di Stefano G., Truini A. (2017). Pharmacological treatment of trigeminal neuralgia. Expert Rev. Neurother..

[77] Rubis A., Juodzbalys G. (2020). The Use of Botulinum Toxin A in the Management of Trigeminal Neuralgia: A Systematic Literature Review. J. Oral Maxillofac. Res..

[78] Cruccu G., Gronseth G., Alksne J., Argoff C., Brainin M., Burchiel K., Nurmikko T., Zakrzewska J.M. (2008). AAN-EFNS guidelines on trigeminal neuralgia management. Eur. J. Neurol..

[79] Montano N., Conforti G., Di Bonaventura R., Meglio M., Fernandez E., Papacci F. (2015). Advances in diagnosis and treatment of trigeminal neuralgia. Ther. Clin. Risk Manag..

[80] Hu Y., Guan X., Fan L., Li M., Liao Y., Nie Z., Jin L. (2013). Therapeutic efficacy and safety of botulinum toxin type A in trigeminal neuralgia: A systematic review. J. Headache Pain.

